# Reperfusion Pulmonary Hemorrhage Following Mechanical Thrombectomy for Sub-massive Pulmonary Embolism

**DOI:** 10.7759/cureus.103167

**Published:** 2026-02-07

**Authors:** John Bajouka, Ghaid Touza, Keyur Patel, Ziad Affas, Stephen Lynch

**Affiliations:** 1 Internal Medicine, Henry Ford Health System, Southfield, USA; 2 Cardiology, Henry Ford Health System, Southfield, USA

**Keywords:** endothelial injury, pulmonary hemorrhage, pulmonary thrombectomy, reperfusion injury, submassive pulmonary embolism

## Abstract

Mechanical thrombectomy is an increasingly utilized intervention for patients with intermediate-risk pulmonary embolism who demonstrate right ventricular strain. While effective at reducing clot burden and improving hemodynamic parameters, the procedure carries a risk of rare post-interventional complications. This case report describes a 75-year-old woman with a history of breast carcinoma who presented with progressive dyspnea and right lower extremity swelling following recent air travel. Diagnostic imaging revealed a sub-massive right-sided pulmonary artery occlusion and severe right ventricular dilatation with reduced systolic function. The patient underwent successful mechanical thrombectomy, resulting in the removal of large, organized thrombi and immediate improvement in pulmonary blood flow. However, the patient developed acute hypoxia shortly after the procedure. Subsequent radiographic imaging showed extensive new consolidation in the right upper lobe. This condition, characterized by alveolar consolidation in previously occluded territories, can radiographically mimic aspiration or procedural trauma. The diagnosis of reperfusion pulmonary hemorrhage was guided by the presence of flow through the pulmonary vasculature and the absence of contrast extravasation on repeat angiography. The patient was managed with supportive care and supplemental oxygen, leading to clinical stabilization and eventual discharge on oral anticoagulation. This case highlights the importance of recognizing reperfusion injury as a distinct clinical entity following mechanical thrombectomy. Clinicians must distinguish this inflammatory and pressure-related phenomenon from direct catheter-induced trauma to ensure appropriate management and avoid unnecessary intervention.

## Introduction

Pulmonary embolism (PE) remains a significant cause of cardiovascular morbidity and mortality, particularly in older adults. While anticoagulation is the standard of care for low-risk cases, mechanical thrombectomy (MT) has emerged as a vital interventional option for patients with submassive PE who exhibit signs of right ventricular (RV) strain or clinical deterioration. The prevalence of submassive PE is notable for its potential to progress rapidly to hemodynamic collapse, necessitating treatments that can quickly alleviate RV pressure overload [1]. Despite its efficacy, the widespread adoption of MT still faces the risk of rare but serious periprocedural complications. Known post-intervention complications include vascular injury, arrhythmia, and hemothorax. One of the most diagnostically challenging complications is reperfusion pulmonary hemorrhage, a phenomenon that can be radiographically indistinguishable from procedural bleeding. Identifying this condition early is essential to avoid unnecessary interventions, such as reversal of life-saving anticoagulation. This case report describes a classic presentation of reperfusion injury following mechanical thrombectomy for a large right-sided PE. Emphasizing this case is critical for clinicians to better recognize and manage the unique radiographic and clinical signature of abrupt flow restoration.

## Case presentation

A 75-year-old woman with a history of stage 2 right breast carcinoma, hypertension, and recent air travel to Texas presented with progressive exertional dyspnea over one week, worsening over the preceding days. She reported right lower extremity swelling extending from the foot to the groin one week prior, which resolved spontaneously. She denied prior venous thromboembolism, congestive heart failure, or family history of thrombosis. Additional symptoms included nonproductive cough, congestion, and right lower rib discomfort. She denied fevers, chills, chest pain, hemoptysis, nausea, vomiting, diarrhea, abdominal pain, dysuria, or calf tenderness. Initially, the patient was hypoxic but hemodynamically stable, requiring two liters of nasal cannula. High-sensitivity troponin resulted at 21 with a repeat of 19; pro-BNP was 1163 pg/mL (Table 1).

**Table 1:** Admission Clinical Data and Laboratory Results bpm: beats per minute; mmHg: millimeters of mercury; ng/L: nanograms per liter; pg/mL: picograms per milliliter; mEq/L: milliequivalents per liter

Assessment	Results	Reference Ranges
Blood Pressure	146/83 mmHg	<120/80 mmHg
Heart Rate	96 bpm	60–100 bpm
Oxygen Requirement	2 Liters per Minute per Nasal Cannula	Room Air (0 L/min)
Arterial Blood Gas		
pH	7.51	7.35–7.45
Partial Pressure of Oxygen (PO2​)	75 mmHg	75–100 mmHg
Bicarbonate (HCO3​)	26.3 mEq/L	22–26 mEq/L
Cardiac Biomarkers		
High-Sensitivity Troponin (Initial)	21 ng/L	0–14 ng/L
High-Sensitivity Troponin (Repeat)	19 ng/L	0–14 ng/L
Pro-B-Type Natriuretic Peptide	1163 pg/mL	<125 pg/mL

**Figure 1:**
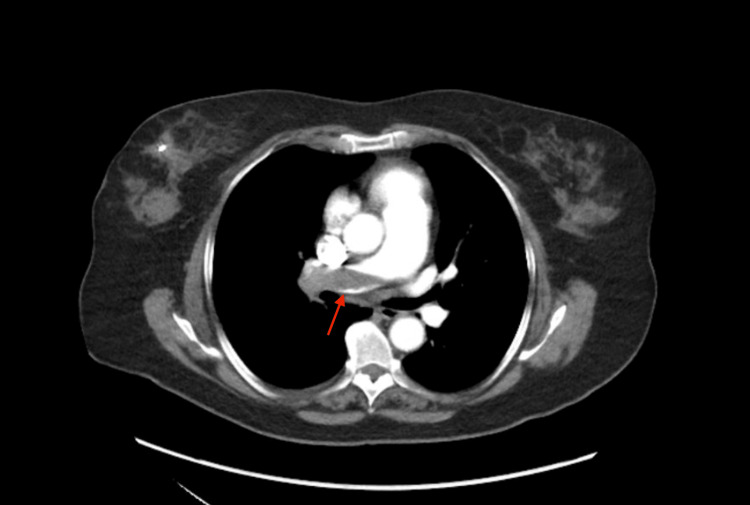
Initial CT Angiography Demonstrating Pulmonary Embolism A large pulmonary embolus in the right pulmonary artery is seen.

**Figure 2:**
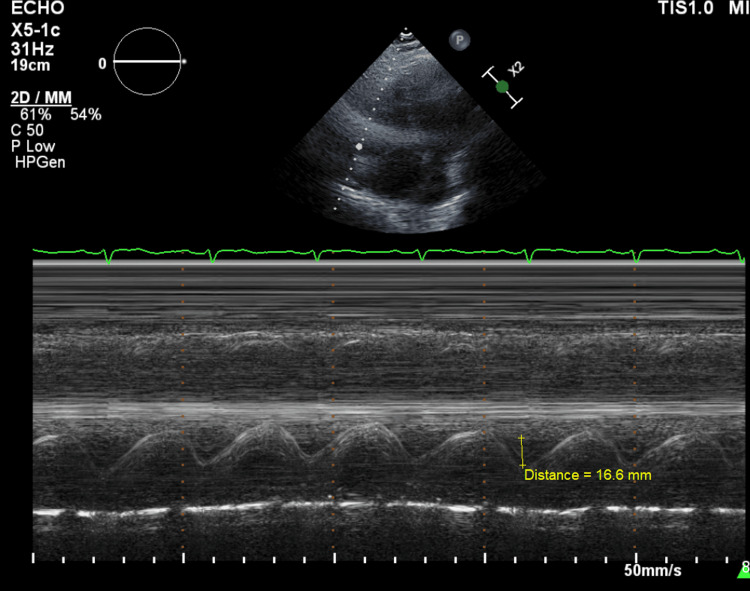
M-Mode Echocardiography from an Apical Four-Chamber View Measurement of tricuspid annular plane systolic excursion (TAPSE) demonstrating a value of 16.6mm indicating right ventricular dysfunction.

The admitting electrocardiogram showed normal sinus rhythm with no signs of right ventricular strain. Computed tomography (CT) angiography to assess for PE showed total occlusion of the right pulmonary artery (Figure 1). Echocardiography demonstrated a dilated RV with reduced systolic function (Figure 2). Given the large clot burden and impaired RV function, the patient was started on a heparin infusion and taken for mechanical thrombectomy. The right anterior basal pulmonary artery, right middle lobar pulmonary artery, and the right main pulmonary artery were evacuated of thrombus with angiographic improvement in flow.

**Figure 3:**
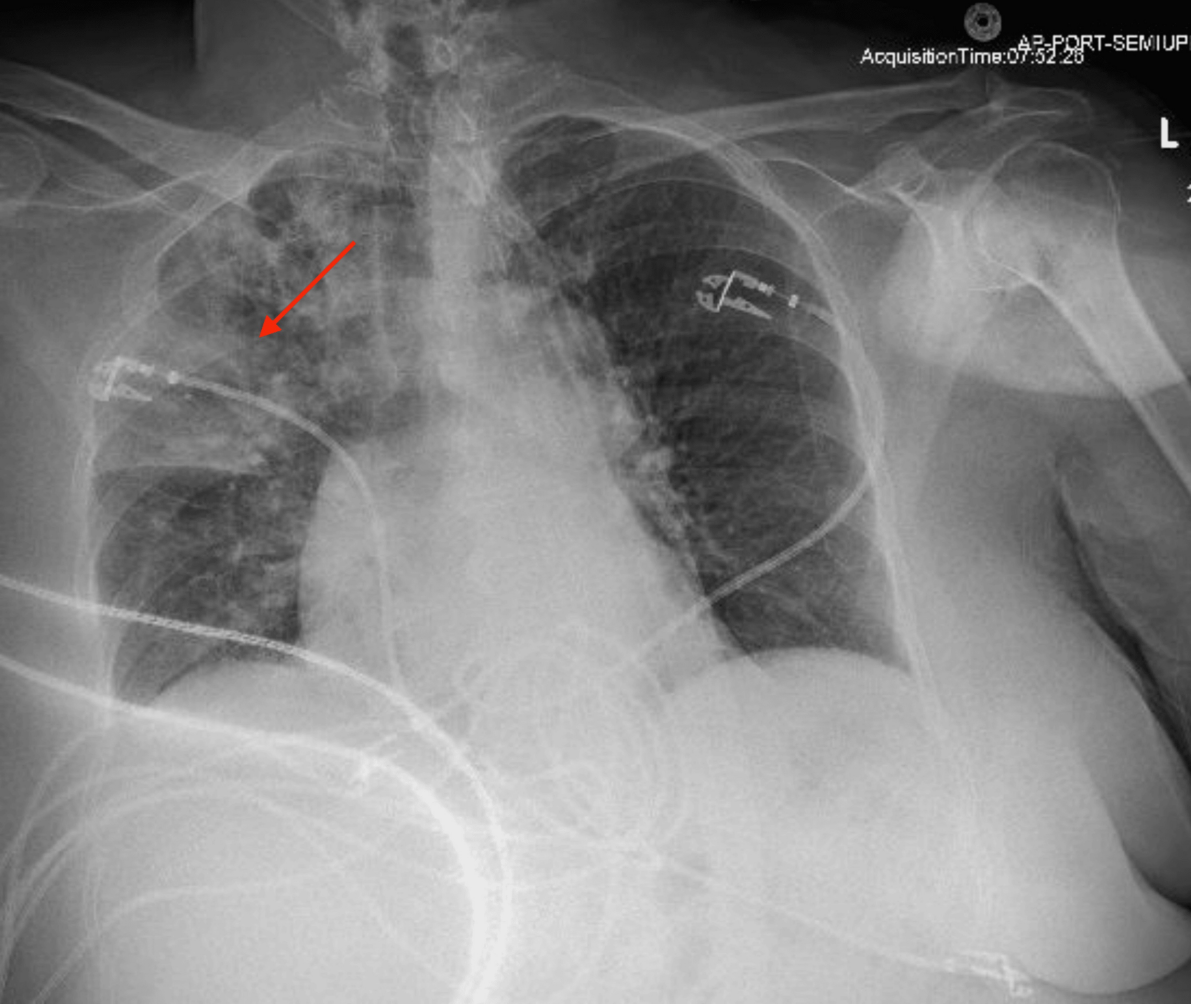
Post-thrombectomy Chest X-ray A large right upper lobe consolidation is demonstrated.

**Figure 4:**
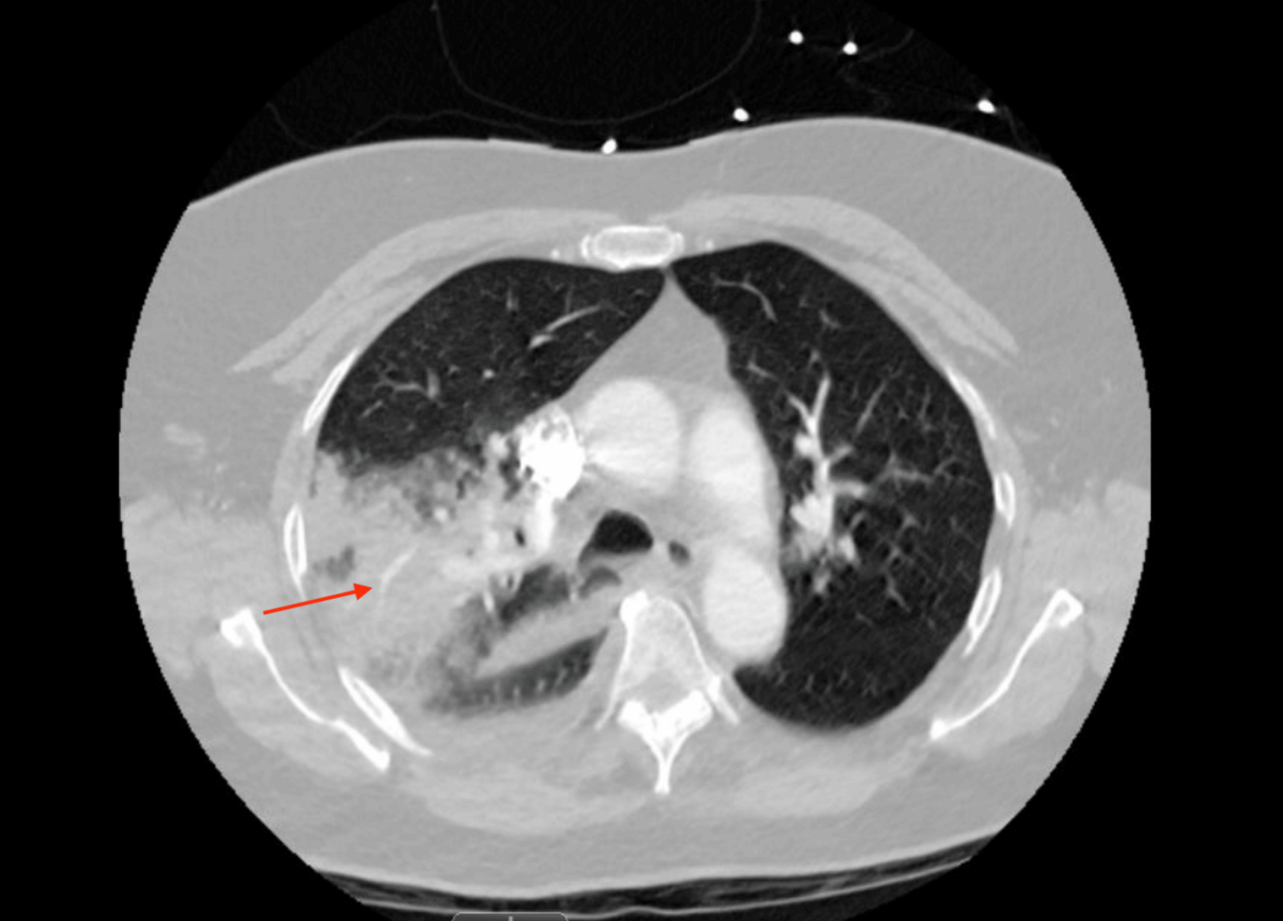
Repeat CT Angiography Post-thrombectomy Interval evacuation of right main and lobar pulmonary arterial emboli. Extensive consolidation within the right upper lobe with restored contrast opacification demonstrating patent blood flow.

Shortly following the mechanical thrombectomy, the patient’s respiratory status declined. Despite being maintained on five liters via nasal cannula, she became increasingly hypoxic, with her oxygen saturation dropping to 89%. This rapid desaturation necessitated an urgent escalation of respiratory support. The patient was transitioned to a high-flow nasal cannula, eventually requiring a flow rate of 35 liters per minute to maintain adequate oxygenation. A chest X-ray was obtained, which showed opacification of the right upper lobe, which was concerning for pulmonary hemorrhage (Figure 3). A repeat CT angiography of the chest demonstrated an extensive right upper lobe consolidation, patchy densities in the middle and lower lobes, small reactive pleural effusion with no contrast extravasation to suggest vascular injury (Figure 4). Due to intact vasculature, the patient was diagnosed with reperfusion injury. 

The patient remained clinically stable. Her hypoxia improved with no evidence of hemoptysis. No further complications were observed. She was monitored, transitioned to oral apixaban, and safely discharged home.

## Discussion

This case illustrates a classic presentation of reperfusion injury following mechanical thrombectomy for an extensive PE. The patient’s clinical course, marked by acute hypoxia and radiographic opacification immediately following successful clot evacuation, highlights a rare but significant complication of advanced PE interventions that clinicians must be prepared to manage.

The pathophysiology of reperfusion injury in the pulmonary vasculature is complex. The primary mechanism involves the sudden restoration of high-pressure flow to chronically hypoperfused lung parenchyma, leading to capillary rupture from abrupt pressure shifts. Furthermore, the rapid reintroduction of oxygenated blood triggers an inflammatory endothelial injury [2]. This process releases reactive oxygen species, including superoxide, hydrogen peroxide, and peroxynitrite, damaging cellular proteins, lipids, and DNA through oxidative stress, triggering lipid peroxidation and opening of the mitochondrial permeability transition pore and pro-inflammatory, increasing microvascular permeability and leading to fluid and red blood cells accumulation into the alveolar space [3].

A nuanced diagnostic challenge highlighted by this case is the interpretation of post-procedural imaging regarding the evacuation of the clot. While mechanical thrombectomy aims for complete debulking, it is common for residual mural thrombus to remain adherent to the vessel walls [4]. Interestingly, repeat angiography or CT may still show "filling defects" that do not represent a failure of the procedure, but rather the altered flow dynamics around residual fragments. Conversely, the restoration of blood flow to a previously occluded segment can sometimes be visualized as "hyperemia" or increased contrast enhancement in the distal parenchyma, which may be mistaken for active hemorrhage if not correlated with the patient's hemodynamic stability. Procedural trauma typically presents with immediate hemodynamic instability, brisk hemoptysis, and clear contrast extravasation on angiography [5]. In contrast, this patient remained hemodynamically stable, had no hemoptysis, and repeat CT angiography confirmed intact vasculature despite the new parenchymal opacities.

Risk factors identified in this patient included a large clot burden, a significant period of ischemia in the right lung, and the rapid nature of mechanical flow restoration. Current guidelines from the American Heart Association emphasize that patient selection for catheter-based interventions should balance the risk of PE-related mortality against procedural complications [6]. Management is primarily supportive, focused on supplemental oxygen and monitoring. While severe cases may necessitate airway protection or temporary anticoagulation reversal, this case reinforces that early recognition through imaging allows for the safe continuation of life-saving anticoagulation while the inflammatory response resolves. Inhaled nitric oxide or prostacyclin may improve ventilation-perfusion matching, and extracorporeal life support may be required when less aggressive interventions fail [7].

## Conclusions

Mechanical thrombectomy remains an effective therapy for sub-massive PE. However, clinicians should recognize reperfusion pulmonary hemorrhage as a rare but potential complication, particularly in cases involving large clot burden and abrupt flow restoration. Early imaging, supportive care, and careful review of procedural angiography are essential for distinguishing reperfusion injury from procedural trauma.
